# Supplementary material for: Multimodal spatial availability: A singly-constrained measure of accessibility considering multiple modes
Source: PLoS One. 2024 Feb 23;19(2):e0299077. doi: 10.1371/journal.pone.0299077 (PMC10889880; doi:10.1371/journal.pone.0299077)
Supplement: Attachment — Submitted filename: Response-to-reviewers_R1.pdf [file pone.0299077.s001.pdf]

PLoS ONE submission: Response to reviewers

2023-11-09

Thank you for for the time and care taken by the editorial team and reviewers in providing feedback to this manuscript. No doubt, the revised version submitted is more articulate than the first. Our responses to all comments from each reviewer are shown below in blue.

Reviewer #1:

The article extends the concept of spatial accessibility and applies it to a fairness analysis of different modes of transportation. The article uses travel data from a week in Madrid as an example to analyse spatial accessibility in the city. However, the article has the following issues:

We appreciate your effort reading our submission and respond in detail to your comments below.

1. Lack of Innovation: The article's improvement on spatial accessibility mainly involves incorporating the ratio of travel frequencies as weight in travel costs. This improvement is relatively minor and the method used does not demonstrate superiority over traditional accessibility approaches.

Thank you for this comment. We would like to bring to your attention the editorial philosophy of PLoS ONE, according to which editors "make decisions on submissions based on scientific rigor, regardless of novelty," (see https://journals.plos.org/plosone/s/editorial-and-peer-review-process). Nonetheless we wish to respond to this comment.

In this paper we extend our earlier work on spatial availability (https://doi.org/10.1371/journal.pone.0278468) for the simultaneous analysis of multiple modes of transportation. You appear to be under the misaprehension that the main difference with accessibility measures is to incorporate "the ratio of travel frequencies as weight in travel costs". This statement is not correct. The main difference is the proportional allocation mechanism that we introduced for spatial availability, and that is extended in this paper to allocate opportunities based on the proportion of travelers by different modes. This is not the same as "weighting the travel cost". In fact, each mode is modelled using its own impedance function, as shown in our empirical example, where we use origin-destination data by mode to estimate travel impedance functions specific to each mode.

While this enhancement may appear minor in your opinion, it is not plainly obvious how spatial availability applies to multiple modes, which is why we believe this paper is needed. We do not dispute that this is an incremental step in the development of a more general method, but it is a step that considerably expands the range of potential applications. Further, we contend that the method has been rigorously developed and demonstrated this using an open, transparent, and reproducible example.

In response to this comment we have edited the paper to more clearly describe the advantages that multimodal spatial availability offers when considering the accessibility to opportunities by different modes. In particular, proportional allocation of opportunities using mode-specific impedance functions means that the travel-cost-advantage of each mode can be analysed. Further, this mechanism ensures that the sum of all spatial availability values for all modes sum up to the total number of opportunities in the region, which is not true for any other type of accessibility measure.

With respect to the "superiority" of the method, a reader of our earlier paper would already be aware of what limitations of accessibility analysis spatial availability aims to address. In this paper we also try to demonstrate throughout the manuscript what our measure does that others can't. Since spatial availability values result from proportional allocation, each value is a proportion of the total number of opportunities. Put another way, we can compare the proportion of opportunities available to users of each mode, to users at

each zone, to users of each mode by zone, and so on, and the values relate directly to the total opportunities in the region. This also allows us to calculate values per capita that serve as benchmark values, as shown in the empirical example.

2. Insufficient Experimental Data: The article uses questionnaire data from a week in Madrid, but lacks basic descriptions of the questionnaire data. Additionally, the daily travel data of approximately 30,000 trips is significantly limited, and there is no description of the criteria for selecting the dates. The typicality of the experimental data is questionable.

Thank you for this comment. To clarify, the empirical data used is not experimental. It is observational, since it is collected using a travel survey conducted by the City of Madrid. Travel surveys are a standard instrument in transportation planning and research, and are conducted in cities around the world. The data we work with represent the most recent and most complete travel survey conducted for the region to date. We are not completely sure where you got the figure of "30,000 trips" (which you consider significantly limited); presumably you are citing the maximum 'opportunity' or 'population' numbers in Figures 2 or 3. The most job-rich TAZs have 30,000 jobs while the least have 1,000 or fewer (Figure 2). In fact, there are 847,574 jobs in the city, which is also the sum of the total spatial availability in our analysis, as well as the number of potential trips to work.

With respect to The "typicallity" of the data, travel surveys are designed to provide information about personal travel on a typical day, usually during a period of maximum demand (i.e., not during the summer vacation). This is standard practice for these surveys, and the one for Madrid is no different in this respect.

3. Lack of Data-Driven Analysis: In the analysis section, a large amount of data is used to analyse people's travel behavior in different areas. The analysis of spatial accessibility only considers the differences in the modes of travel mentioned earlier. The analysis results are somewhat one-sided, and examining the indicator from multiple perspectives would provide a more objective view. It is recommended to expand the dimensions of the analysis.

Thank you for this comment. It is somewhat puzzling that "30,000 trips" would be significantly limited (as per your comment #2), and at the same time be "a large amount of data". We find that this comment in particular is not actionable due to its vagueness. There are no meaningful responses to "somewhat one-sided" when "somewhat" and "one-sided" do not quantify or refer to a particular side. We studied the modes available in the region, and examined the results from the perspective of each mode. What other perspectives do you suggest? What dimensions should be explored?

Despite this comment being nonactionable, we believe that with the additional detail added to the manuscript to address your and other reviewers' comments, that overall clarity has been improved.

4. Disorganized Format: The article's methodology section contains numerous formulas and variables, but the definitions of these variables are unclear and difficult to read. There are also numerous formatting errors, and Table 1 is disorganized and unappealing. It is recommended to revise these issues.

Reviewer #2 identified some specific formatting issues and we fixed them. Otherwise we proofread the paper and hopefully did not leave a typo behind. We also made sure Table 1 starts on a new page, when it runs across pages the formatting resulted in some disorganization.

Thank you again for your efforts trying to review our submission.

Reviewer #2:

This paper has sound mathematical foundations and allows to answer its research question in an elegant way (how to measure competition for e.g. jobs based on spatial accessibility?). I also really appreciated the fact that the paper was very didactic. Still, I have concerns about the relevance of the paper for future research.

Thank you for your thoughtful review of our paper. Your comments were very helpful to improve the clarity of the research.

More specifically, the new measure proposed by the authors has clear limitations: i) it focuses on the competition for jobs and is not useful for studying access to non-competitive or semi-competitive resources such as amenities.

Thank you for this comment. We would begin by noting that there are many types of opportunities that are mutually exclusive due to competition. For this paper we focused on jobs, but there are many others, such as beds at hospitals, seats at schools, and so on. Thus, even if the measure was applicable only to exclusive opportunities, there are numerous applications to choose from.

That said, we have grappled for some time with the question of what types of opportunities are best analysed using spatial availability. Our current thinking on this matter, after much consideration, is that in practice every type of opportunity, even when not clearly exclusive, is subject at least to congestion or capacity constraint. For instance, green spaces are often considered non-competitive, however, standards for the provision of such amenties are provided in the form of units of amenity per capita. For example, a case could the Ile-de-France region, a jurisdiction that suggested in a major planning document of 2013 that at the municipal level at least $10m^2$ of public green space should be supplied per inhabitant (Liotta et al. 2020). But green spaces are not evenly distributed, which means that who has access to them hinges on where they are and how easy is to reach them. Formulating the provision of amenities in these terms in not rare. For example, Natural England recommends an Accessible Natural Greenspace Standard such that the minimum supply of space is one ha of statutory Local Nature Reserves*per thousand population*[1] . Similarly, the World Health Organization (cited in OECD, 2013) recommends that cities provide a minimum of 9 m^2 of green area per inhabitant[2] . For our purposes, standards of this type translate into "how much of this resource is available to one individual that has not been claimed by anyone else?". Green spaces often have large capacities, but they still have a capacity, and it is not the same for a person to have access to $5m^2$ of *uncongested* green space than to $15m^2$. This difference is in fact a matter of justice (Lara-Valencia and García-Pérez 2015; Liotta et al. 2020). Constraining accessibility is in this way a useful way to evaluate the congested availability of any type of opportunity. As standards are emphasized in the planning literature, in particular for fairness in transportation (see Martens and Golub 2021), spatial availability analysis can be used to assess standards. We are convinced that as other researchers discover this new approach to measuring accessibility many other applications will be found.

ii) it doesn't allow to study absolute gains or losses in accessibility from public transportation infrastructure improvements or changes.

Great point. Spatial availability can certainly be used to capture absolute gains and losses. In fact, logically, the gains and losses produced by using a competitive and constrained measure allows for a clear interpretation. This would require the analyst to estimate the accessibility before and after some change to the land use or transportation system. In this paper our empirical application is a single scenario to serve as proof of concept, but in future research we intend to use spatial availability to analyse changes in the size of Madrid's Low Emissions Zone implementation from a modal and socio-economic equity perspective.

iii) the authors do not allow for modal shifts: they assume that the transport mode choice of households is fixed and cannot evolve due to e.g. transport infrastructure changes.

This is an excellent point. Accessibility measures, including spatial availability, are not meant to function as modal split models, but they are certainly amenable to analysis of changes in the accessibiity landscape

[1]see https://redfrogforum.org/wp-content/uploads/2019/11/67-Nature-Nearby%E2%80%99-Accessible-Natural-Greenspace-Guidance.pdf

[2]see https://doi.org/10.1787/9789264191808-en

if different modal shares are used as inputs. This is similar to the case of destination choice: accessibility measures do not model this, but changes in destination choices can be incorporated via how they affect the impedance function. In terms of how this could be implemented, the process can be sketched as follows: the results of a modal split model are used to estimate new modal shares, which in turn are used to recalculate spatial availability. The new values then can be compared to the baseline scenario. In other words, the framework for spatial availability is sufficiently flexible to take in not only mode-specific travel impedance functions, but also the proportions of the populations using each mode.

These points limit the relevance of the new accessibility measure. The authors should, at least, specify these limitations early in the paper. The introduction should start by stating the precise research question the new accessibility measure is seeking to answer as well as its limitations). They should also justify, based on the literature, that competition for jobs is a key determinant of job market outcomes, and that there is strong inertia in mode choices.

Your comments have been very helpful to improve the clarity of the paper, as well as the scope of what we do, as well as directions for future research. For example, we now note in the "Discussion and conclusions" that "our example dealt with differences in travel by mode only, but it is possible to think of the intersection between mode of travel and different types of travelers. This would expand the number of sub-populations in the analysis from, say, $m = M$ (modes) to $m = M \cdot Q$ (modes times population segments), each with their own characteristic impedance function. Evaluations of this kind will be especially relevant as LEZ are implemented in cities globally, and the question of their impact on disadvantaged populations who have become mobility-restricted increasingly come to the fore (De Vrij and Vanoutrive 2022; Verbeek and Hincks 2022; Liotta 2023)."

Finally, the writing of the paper, and particularly of the abstract and introduction, should be improved. The abstract could state the broader relevance of the topic and summarize the results of the case study on LEZs. The introduction should start more directly by introducing the research question and its relevance and describing the new measure and its limitations.

We have now done this. In the original version of the abstract and introduction we gave the misleading impression that we would analyse changes in the system, when in reality our intent was to demonstrate the application of the measure in an empirical example. We do plan to study changes in the system, but this requires more work than can be presented in a single paper, partly for the reasons that you identified above (modal shifts and the calculation of spatial availability for two different scenarios, as well as the analysis of the differences between them). We plan to do this in a future paper focused on the policy instead of the presentation of a new method.

Thank you again for your thoughtful comments and suggestions to improve the paper.

Minor comments:

i) what are the summary statistics p10 (car: 36 min, transit: 55 min,...)? I assume they correspond to the mean.

Updated! Apologies, car: '36 min' corresponds to a mean of 36 minutes and then within the brackets additional descriptive statistics (minumum value, maximum value, etc.).

ii) I also have identified a few formatting issues (e.g. Fcij p10 and 4.72km2 p 8).

Fixed! Thank you.

Reviewer #3:

This manuscript extended the authors' previous work spatial availability measure, which is a type of location-based accessibility measure that is both constrained and competitive compared to Hansen-type measure and Shen-type accessibility measure, into a multimodal framework. The new measure, multimodal spatial availability, strengthened the constrained (or finite) nature of opportunities, and the competitive nature among multimodal accessibility resulting from this constraint through a synthetic example and an empirical example of the LEZ in the city of Madrid. In conclusion, the authors demonstrated one restriction had impacted the spatial availability of opportunities for other modes using and proposed potential future uses in policy planning scenarios.

In general, the manuscript was logical and well-structured. The research problem was well defined. The data were available and quite supported the conclusion. The statistical analysis performed appropriately.

However, there are some issues:

Major issues:

Please demonstrate whether "car/motor & transit" and "bike & walk" are comparable or whether they are in an actual competitive relationship? For example, if I work 3km from where I live, maybe I will never choose to take a transit, I will always walk or ride. But if I work 20km from where I live, walking or riding to work seems impossible for me, I have to drive or take public transportation. Car/motor and transit can be in competitive relationship and people can choose which one they prefer, but not choose between motor and walk. This issue will also have an impact on the results of the research.

This is an excellent comment. The short answer is that whenever a destination can be reached by more than one mode, users of those modes are in competition for the opportunities there.

In this revision we tried to improve the discussion to make this point more clear. The impedance functions for all four modes are not the same. They describe the travel behaviour of commuters as informed by the 2018 travel survey. To follow your example, someone who cannot walk to work because their job is 20 km from where they live, will not compete for that job against people from their same origin who do walk. However, if the place where their work is can be reached by anyone who can walk from other origins (say, someone who lives closer to that destination), they would be in competion for the same opportunity.

Futhermore, average travel times for car/motor and transit are longer than bike and walk. All people don't have access to all options - completely true. But the travel impedances reflect this real travel at an aggregate based on all the trips for a mode. And on average, it is assumed that people at each origin that take a mode to a destination are in direct competition for opportunities (as opportunities are finite) – and a part of the competition is defined by the mode-specific impedance function (the second part is the population balancing factor). This assumption, that all populations, no matter their mode, are competing for the same finite set of opportunity, is part of spatial availability. We've made an effort to make this more clear in the text.

Minor issues:

1. As for Fig 2 and Fig 3, please indicate the meaning of the gray color blocks in illustration.
2. Please change the color scheme of fig 2. The red color scheme makes the LEZ centro area boundary, which is also in red, not visible.

We have updated the captions of the figures to reflec the meaningof grey colour blocks and the colour scheme of all Figures. We've also updated the labels on the legends to make them more interpretable.

References

De Vrij, Eva, and Thomas Vanoutrive. 2022. "'No-One Visits Me Anymore': Low Emission Zones and Social Exclusion via Sustainable Transport Policy." 2022. https://www.tandfonline.com/doi/epdf/10.1080/1523908X.2021.2022465?needAccess=true&role=button.

Lara-Valencia, Francisco, and Hilda García-Pérez. 2015. "Space for Equity: Socioeconomic Variations in the Provision of Public Parks in Hermosillo, Mexico." Journal Article. *Local Environment* 20 (3): 350–68. https://doi.org/10.1080/13549839.2013.857647.

Liotta, Charlotte. 2023. "What Drives Inequalities in Low Emission Zones'impacts on Job Accessibility?" In. Virtual. https://doi.org/10.17605/OSF.IO/9VQU7.

Liotta, Charlotte, Yann Kervinio, Harold Levrel, and Léa Tardieu. 2020. "Planning for Environmental Justice - Reducing Well-Being Inequalities Through Urban Greening." Journal Article. *Environmental Science & Policy* 112: 47–60. https://doi.org/https://doi.org/10.1016/j.envsci.2020.03.017.

Martens, Karel, and Aaron Golub. 2021. "A Fair Distribution of Accessibility: Interpreting Civil Rights Regulations for Regional Transportation Plans." Journal Article. *Journal of Planning Education and Research* 41 (4): 425–44. https://doi.org/10.1177/0739456x18791014.

Verbeek, Thomas, and Stephen Hincks. 2022. "The 'Just' Management of Urban Air Pollution? A Geospatial Analysis of Low Emission Zones in Brussels and London." *Applied Geography* 140 (March): 102642. https://doi.org/10.1016/j.apgeog.2022.102642.
