# Supplementary material for: Multimodal spatial availability: A singly-constrained measure of accessibility considering multiple modes
Source: PLoS One. 2024 Feb 23;19(2):e0299077. doi: 10.1371/journal.pone.0299077 (PMC10889880; doi:10.1371/journal.pone.0299077)
Supplement: Attachment — Submitted filename: Response-to-reviewers_R2.pdf [file pone.0299077.s002.pdf]

PLOS ONE submission: Response to reviewers

2024-01-26

Thank you for for the time and care taken by the editorial team and reviewers in providing feedback to the second round of revision to the manuscript. No doubt, the further revised version submitted is more articulate than the first. Our responses to comments to Reviewer #2 are shown below in blue. Reviewer #3 and #1 did not have any additional comments.

Reviewer #2:

I appreciate the fact that the authors have largely revised the paper to answer the reviewers' comments. Still, I feel like some of my comments have been insufficiently addressed in the revised draft, as detailed below.

Thank you: comments provided by all reviewers were exceptionally helpful in clarifying the manuscript. We address the outstanding three comments and additional minor comments in this response letter.

The authors have adequately addressed my comment on the fact that their new measure is not useful for studying access to non-competitive resources in the revised introduction.

Thank you, we appreciate your understanding.

My comment on the fact that the new measure doesn't allow to study the absolute gains or losses in accessibility from public transport or land use system changes has inadequately been accounted for. First, what your new measure can or cannot do remains unclear to me: for instance, how would an improvement in the transport sector that benefits all transport modes and locations uniformly, but still reduces all transportation times in the city, be accounted for? Second, I do not see where this point is discussed in the paper.

This is a great question, and we think we know the answer. We interpreted your comment initially as to mean changes in accessibility, and we responded accordingly. Your example makes it clear that you had something else in mind. The answer to your question can be found in point 2 in the conclusion of Soukhov et al. (2023) (p. 25) where it says "Shen-type accessibility can be used to compute the availability of jobs (the rate and the absolute values if the original definition is corrected), however, if the analyst is interested in internal values and secondary analysis of the results, spatial availability should be considered". The internal values referred to there are the estimates of trip length/duration/cost associated with the spatial availability of opportunities; the secondary analysis, in turn, referred to estimating the system-wide cost of competitive accessibility. To use your example, suppose that there is a change in the transportation system that is uniform: accessibility does not change but travel times are shorter for every mode. As Soukhov et al. (2023) demonstrate in the section *Why does proportional allocation matter?* (p. 15), a difference between spatial availability and unconstrained accessibility measures, is that spatial availability allocates the actual number of opportunities to each origin. As a result, the estimates of travel time can be used to quantify the system-wide impacts of spatial availability. In your hypothetical example, spatial availability would remain constant, but the system-wide cost of travel, as calculated from the intermediate values (i.e., the detailed table of opportunities allocated to origins by origin-destination pair), would decrease.

My comment on modal shift has partially been accounted for. A key question is whether people can shift transport modes easily or not: indeed, it seems to be that your new accessibility measure, based on competition, does only make sense in case people cannot easily shift transport modes.

Thank you for your comment. For the empirical example we use data collected at least one year *after* the introduction of Madrid LEZ. We then make the (hopefully reasonable) assumption that all modal shifts caused by LEZ have already taken place. We could (if we had the data) calculate spatial availability using

the mode shares *before* the introduction of Madrid LEZ. Here, however, we are not conducting a before/after comparison of the policy, but only of the spatial availability already in the context of LEZ. In other words, our discussion is based not on a comparison over time, but a comparison over space. We have further enhanced the results to make this interpretation more clear.

You partially answer that question in the paragraph starting l46 by explaining why some populations, e.g. children, elderly, or single parents, might have different characteristics in terms of transportation choice and mode use. Still, in the case of the Madrid LEZ, you do not explain why private cars and public transport users are in competition. For instance, you state that "restrictions to travel by car leave more spatially available opportunities for non-car-users" (l585).

Our discussion of competition is not based on hypothetical modal shifts caused by LEZ, but rather on the comparison of spatial availability after LEZ over space. Fig. 6 in the paper shows that bike and walk consistently have fewer jobs available than their share of users in the population. But, they receive a larger share *within* the boundaries of Centro, where the number of cars that can enter is limited by the LEZ policy. In other words, the restriction, which is what distinguishes Centro from the boundary of the M-30 or the whole region, seems to open up a window of opportunity for jobs to become available to cyclists and pedestrians.

But it seems to me that the LEZ aims at promoting a modal shift toward less polluting cars, public transport, or active modes rather than favoring public transport users?

No question that promoting a modal shift is part of the policy. However, as we noted above, we assume that the data already incorporates said shift. Given the modal shares, our analysis indicates that the winning mode within the boundaries of LEZ is transit (Fig. 6).

Relatedly, your measure does not make any difference between car users that can easily shift to public transport, and car users that cannot.

You are absolutely right. That said, the job of allocating users to various modes is not the job of our measure, but the job of a mode split or mode choice model. The scope of our measure is to allocate opportunities to users by mode. Given changes in the shares of different modes, the measure can be calculated to examine the spatial availability under those conditions.

My other comments have been accounted for, and the writing of the paper has largely been improved.

Finally, I have a few additional minor comments:

Fig 5: the boundaries of the LEZs, and in particular of the LEZ centro, are really hard to see.

There is a typo l56: "The paper rest of the paper"

Thank you bringing these comments to our attention. We have changed the colours and slightly increased the size of the spatial borders for LEZ Centro and M-30 on all figures (now the borders are Pink and Purple instead of Light and Medium Grey) to improve visibility. We have also corrected the identified typo. Additionally, we have improved phrasing and corrected grammatical errors throughout the manuscript.

We wish to thank you for your thoughtful and constructive comments throughout the revision process so far. A sticking point is the shift in modes. In this respect, we would like to reiterate that our measure is not intended to do the things that modal split or mode choice models are supposed to do. Our empirical example is cross-sectional, and we believe bypasses the question of modal shifts while still giving useful information about the spatial distribution of opportunities and the ability of different modes to reach them. We would argue that the materials presented here are already an advance over existing methods–even in the case when accessibility remains constant but the cost of travel declines uniformly. Still, we wish to acknowledge that the use of multimodal spatial availability in over-time comparisons would benefit from being paired with a modal split or mode choice model, something that we are prevented from trying in our empirical example due to data limitations.

References

Soukhov, Anastasia, Antonio Páez, Christopher D. Higgins, and Moataz Mohamed. 2023. "Introducing Spatial Availability, a Singly-Constrained Measure of Competitive Accessibility | PLOS ONE." *PLOS*

ONE, 1–30. https://doi.org/https:// doi.org/10.1371/journal.pone.0278468.
